# Supplementary material for: A computational approach to measure the linguistic characteristics of psychotherapy timing, responsiveness, and consistency
Source: Npj Ment Health Res. 2022 Dec 2;1:19. doi: 10.1038/s44184-022-00020-9 (PMC10956022; doi:10.1038/s44184-022-00020-9)
Supplement: Supplementary file 1 — Supplementary materials [file 44184_2022_20_MOESM1_ESM.docx]

## Supplementary materials

[Supplementary materials 1](#_Toc110862668)

[Supplementary notes 2](#_Toc110862669)

[Dataset 2](#_Toc110862670)

[Phase 1: Feature generation 3](#_Toc110862671)

[Therapist tactics 3](#_Toc110862672)

[Paralinguistic style 4](#_Toc110862673)

[Phase 2: Feature implementation 4](#_Toc110862674)

[Evaluating whether therapist speech is dynamic 4](#_Toc110862675)

[Evaluating whether therapist speech is responsive 5](#_Toc110862676)

[Supplementary Figure 1. Single therapy session language features 6](#_Toc110862677)

[Supplementary Figure 2. Frequency of temporal associations 7](#_Toc110862678)

[Supplementary Figure 3. Directed temporal associations 8](#_Toc110862679)

[Supplementary Table 1. Comparison of language features between quintiles 9](#_Toc110862680)

[Supplementary Table 2. Features considered but not selected for study 10](#_Toc110862681)

##

## Supplementary notes

### Dataset

In the original clinical randomized trial from which our dataset was derived, college counseling centers self-selected into a study comparing an “external expert consultation model” vs. “train-the-trainer” model for improving adherence to interpersonal psychotherapy (IPT), an evidence-based treatment for depression and eating disorders. Counseling centers with a staff member willing to serve as study director and at least three interested therapists were eligible for the study and were cluster-randomized to one of the two treatments. Audio of each therapy session in the study was recorded for the purpose of evaluating treatment as usual vs. therapist fidelity to IPT. We use the audio recordings from the treatment as usual population (i.e., what students would get under normal treatment conditions) for a secondary analysis in our study. We refer the reviewer to Wilfley et al (2018) and Wilfley et al (2020) for details on the original trial protocols for sampling patients.

In total, 855 audio recordings of psychotherapy sessions across 189 unique patient-therapist dyads and 105 unique therapists were recorded in the original study. Due to grant budgeting constraints which prohibited manual transcription of all 855 audio recordings, we used a convenience sampling approach to select 98 audio recordings from these 855 originally recorded sessions. We first randomly sampled 78 unique therapists from the 105 therapists in the original dataset in order to maximize the diversity of therapists within our sample. For each of these therapists, we selected one therapist-patient dyad and, from all of the sessions available for that dyad, selected a single session uniformly at random from those available (e.g., for a therapist-patient dyad with two total sessions, the first was just as likely to be sampled as the second, but only one of these two was sampled). These 78 therapist-patient dyads formed our primary sample.

Subsequently, in order to analyze within-therapist language consistency, we also generated a secondary sample. From the 78 unique therapists in our primary sample, we randomly selected 20 therapists that had at least one other patient in the original trial dataset. A second session from a unique patient (i.e., a patient not already in the primary sample) was selected for each of these 20 therapists to form our secondary sample. These 98 recordings comprising our primary and secondary samples were then sent for human transcription (see Miner et al., 2020).

For feature extraction, including the identification and counting of lexicon-specific matches within a sentence, transcribed text was converted to lowercase and punctuation other than apostrophes (e.g., “it’s” or “you’re”) were removed. For details of our transcription process see prior work (Miner et al., 2020)^14^.

### Phase 1: Feature generation

#### Therapist tactics

*Active Listening*: One approach is to express understanding through simple utterances such as ‘yes’, ‘uh-huh’, or ‘mmhmm’. These utterances are often used as continuers, statements which interrupt the patient but are meant to signal the patient should continue speaking. Hedging is a way for the therapist to state back to the patient something they heard or believe but in a way that invites patient feedback or re-interpretation (e.g., ‘it sounds like’, ‘maybe’). Hedging and checking for understanding are related to listener skill in non-therapy settings.

*Non-judgmental Stance*: By using more balanced and contextual language (e.g., ‘sometimes’, ‘often’, ‘rarely’), the therapist attempts to model a more adaptive speaking and thinking style. Absolutist language, which has not been assessed previously in therapist language, is related to anxiety and depression in non-therapy settings^87^.

#### Paralinguistic style

We measured the seconds taken by each therapist per talk turn, with talk turn boundaries delineated by a change in speaker in the transcript. Because time in each session was recorded by human transcribers at the level of seconds, some of the therapists’ talk turns (8,978 or 0.19%) had an unspecified length of time less than one second. For the purpose of calculating words per second and seconds per talk turn statistics, we imputed the length of these therapist utterances to be 0.5 seconds. We additionally clipped/winsorized the number of seconds for each talk turn to be no more than 120 seconds (2 minutes) and the words per second in each talk turn to be no more than 5 words per second (5 words per second, or 300 words per minute, is approximately the rate of speech of professional auctioneers). Less than 0.1% of talk turns were affected by either form of clipping. We additionally measured therapists’ rates of speech by dividing the number of therapist-spoken words by the amount of time that the therapist spoke, as indicated by the time stamps in the transcripts. In addition to measuring paralinguistic features of the therapist independent of the patient, we also measured the therapist-to-patient ratio of both seconds taken per talk turn and words spoken per second. Including these ratios provides insight into whether the therapist was speaking faster or slower than the patient, as well as taking more time in each talk turn compared to the patient.

### Phase 2: Feature implementation

#### Evaluating whether therapist speech is dynamic

Quintile values were given as the midpoint of the quintile bin (i.e., the independent variable value representing the first quintile was 0.1 for all therapists, 0.3 for the second quintile, and 0.9 for the last quintile). The interpolated feature values (dependent variable) were centered and scaled so that temporal trends from distinct features could be meaningfully compared side by side (see Figure 1a).

#### Evaluating whether therapist speech is responsive

While the analyses of statistical differences between therapist and patient language features within and across time can yield provocative hypotheses about the way in which therapist and patient language are related, such findings are associative and not causal. The temporal structure of our data allows for more robust inference compared to purely associational approaches (because we can reasonably assume that only past patient and therapist language influence present therapist language at any point in the psychotherapy session), but the observational nature of our data would mandate several assumptions to make the discovered associations causally valid. As we cannot necessarily validate these assumptions, we restrain from making any causal claims. These assumptions include causal sufficiency (that all causal drivers are observed in the data), faithfulness (that all observed conditional independencies are encapsulated within the learned graphical structure), and stationarity (that the distribution of the time series in consideration does not change over time). While the first two assumptions (causal sufficiency and faithfulness) are difficult or impossible to validate, especially in the absence of strong and validated theory characterizing causal mechanisms, we explicitly tested against nonstationarity for each feature and removed from our analysis those subjects whose preprocessed language features exhibited nonstationarity.

More specifically, we preprocessed our data via differencing, i.e., by representing the therapist (or patient) language features associated with a talk turn at time *t* as the raw language feature at time *t* minus the raw language feature at time *t* - 1. This approach is commonly employed in temporal causal modeling to achieve stationarity and preserves the intuition of what we would hope to measure via our causal inference approach (i.e., “do sudden increases in a particular patient language feature cause associated increases or decreases in corresponding therapist language features?”). Using the Augmented Dickey-Fuller (ADF) unit root test and Kwiatkowski-Phillips-Schmidt-Shin test for stationarity, we tested the time series distributions of each patient and therapist’s language features to assess whether they were stationary. Out of 2496 language feature distributions tested (16 language features across 78 unique sessions, with one patient and one therapist in each session), only two specific language feature distributions were identified as being nonstationary, testing at confidence level α = 0.05. These sessions were excluded from the temporal association discovery analysis.

## Supplementary Figure 1. Single therapy session language features

**
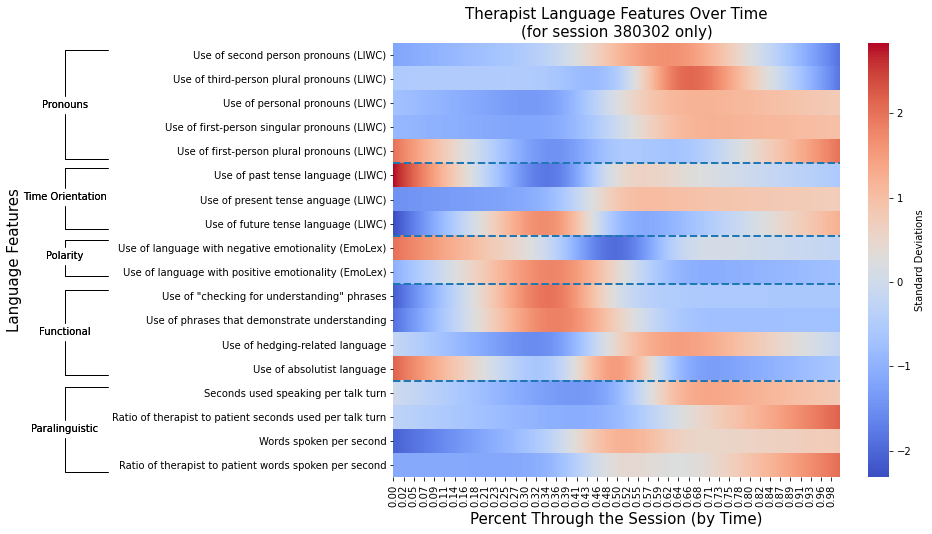

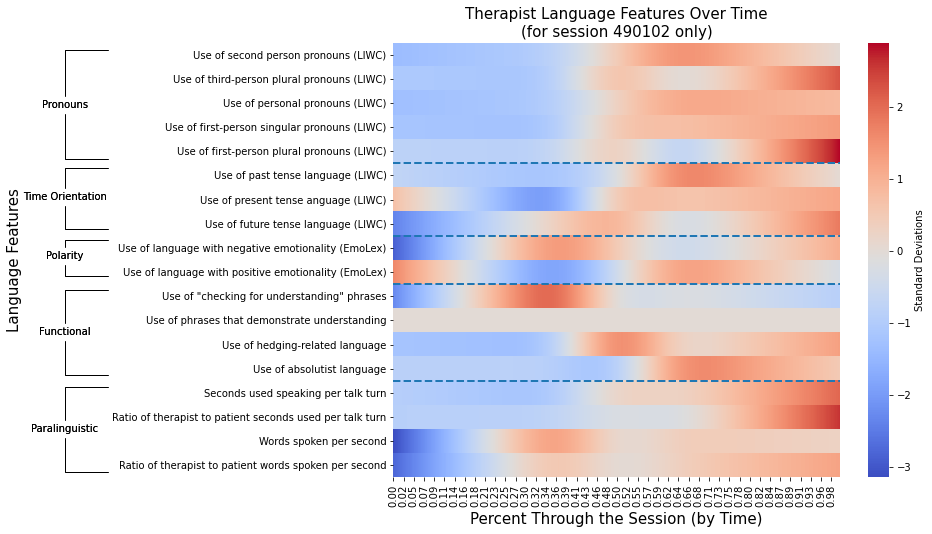
**

**
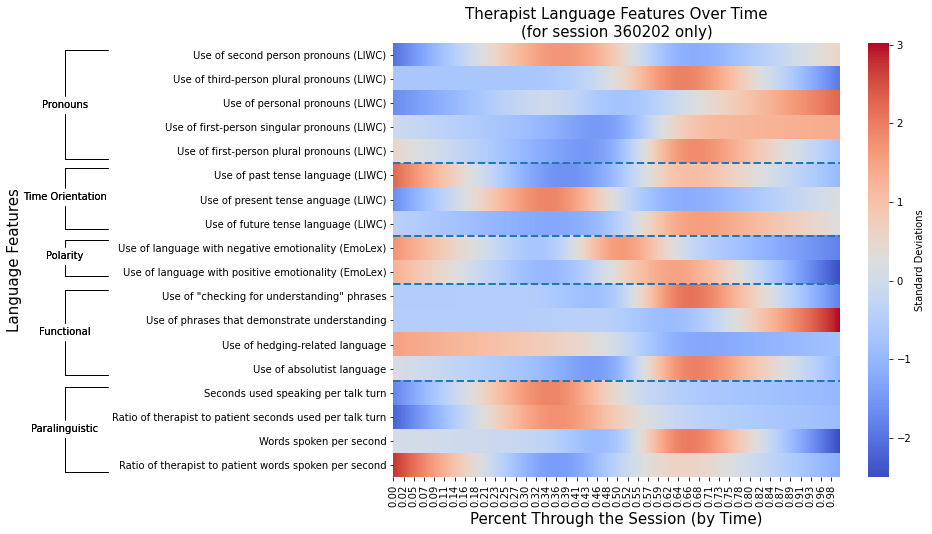

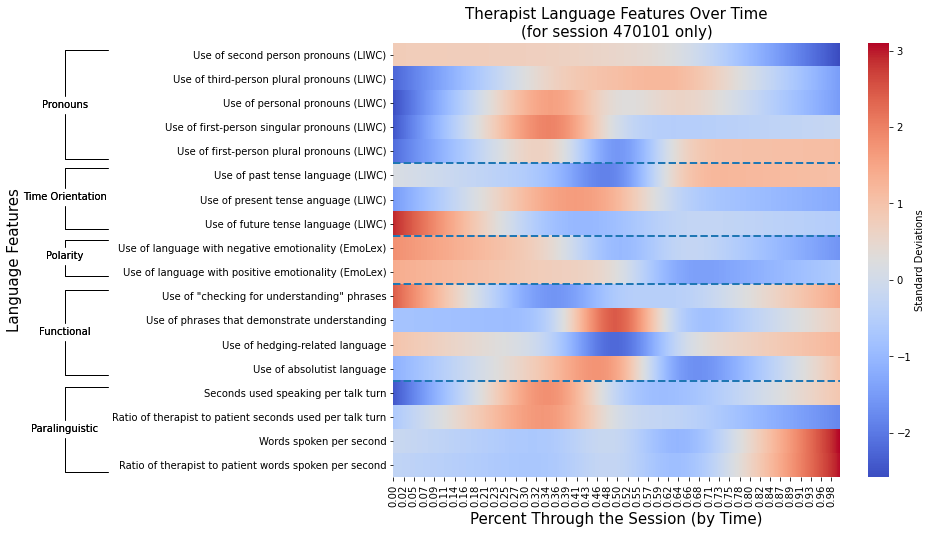
**

**Supplementary Figure 1:** The four figures present the same analytic approach as Figure 1 in the main manuscript, broken out into four single therapy sessions from four individual therapists.

##

## Supplementary Figure 2. Frequency of temporal associations


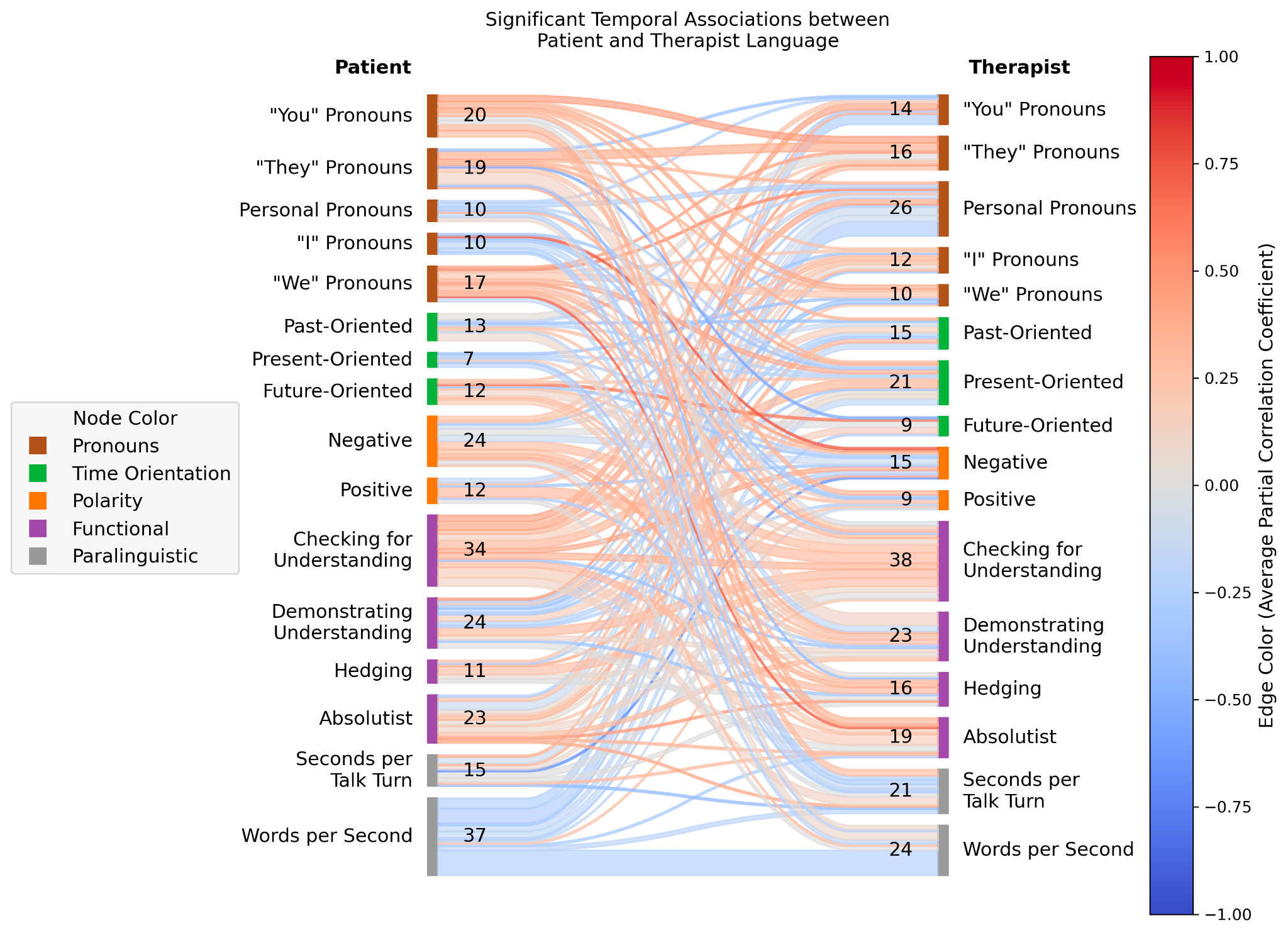


**Supplementary Figure 2:** The frequency with which certain temporal associations between patient language features and subsequent/accommodating therapist language features emerged, across all sessions. Compare to Figure 2 which illustrates a subset of edges, each of which appeared in at least four unique sessions.

##

## Supplementary Figure 3. Directed temporal associations


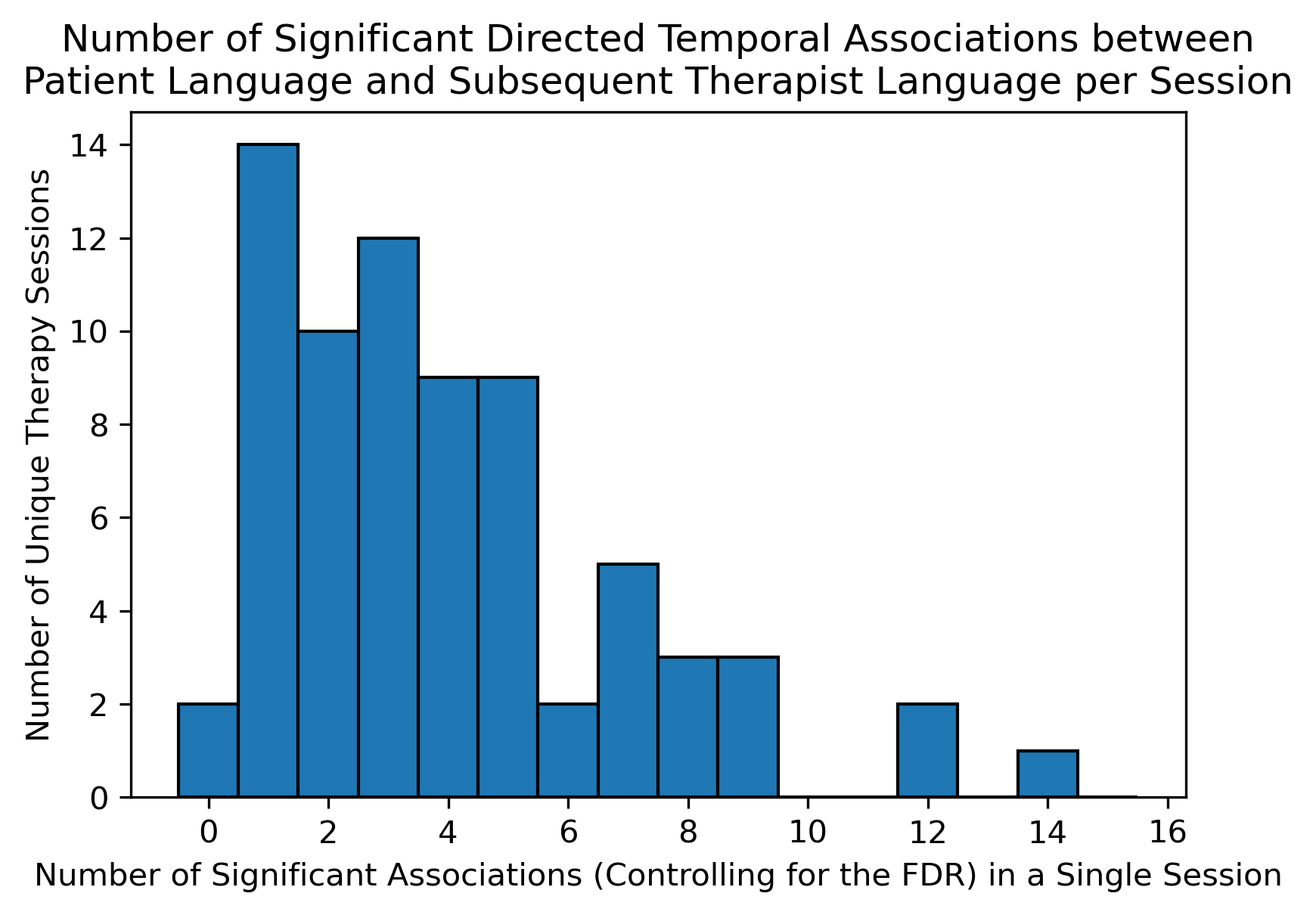


**Supplementary Figure 3:** The number of temporally dependent links between patient language and subsequent therapist language, across all sessions. For example, two sessions exhibited no significant associations and one session exhibited 14.

##

## Supplementary Table 1. Comparison of language features between quintiles

|  | **Q1 Therapist “Q1T”** | **Q5 Therapist “Q5T”** | **Q1 Patient “Q1P”** | **Q5 Patient “Q5P”** | ***P*(Q1T = Q5T)** | ***P*(Q1P = Q5P)** | ***P*(Q1T = Q1P)** | ***P*(Q5T = Q5P)** |
| --- | --- | --- | --- | --- | --- | --- | --- | --- |
| **2nd Person Pronouns** | 0.074808 (0.070093, 0.079978) | 0.080765 (0.076676, 0.085386) | 0.009968 (0.008233, 0.011619) | 0.018042 (0.015310, 0.020881) | 1.88E-02 | 1.36E-05 | 4.57E-27 | 2.56E-26 |
| **3rd Person Plural Pronouns** | 0.004544 (0.003235, 0.006257) | 0.005576 (0.004510, 0.006787) | 0.006948 (0.005755, 0.008305) | 0.005342 (0.003947, 0.006890) | 3.54E-02 | 1.24E-02 | 4.96E-04 | 2.84E-01 |
| **All Personal Pronouns** | 0.118178 (0.111600, 0.125256) | 0.149979 (0.143775, 0.156455) | 0.155274 (0.149702, 0.160996) | 0.166119 (0.159442, 0.172862) | 3.86E-10 | 2.08E-02 | 3.13E-12 | 4.50E-04 |
| **1st Person Singular Pronouns** | 0.023841 (0.020646, 0.027122) | 0.041500 (0.037159, 0.046123) | 0.113585 (0.107188, 0.119677) | 0.120268 (0.112819, 0.128395) | 1.93E-08 | 2.32E-01 | 6.25E-27 | 2.78E-24 |
| **1st Person Plural Pronouns** | 0.007180 (0.005458, 0.008778) | 0.015046 (0.012884, 0.017540) | 0.006715 (0.005271, 0.008253) | 0.007142 (0.005417, 0.008852) | 8.25E-08 | 9.89E-01 | 6.26E-01 | 1.93E-07 |
| **Past Focus** | 0.041586 (0.037730, 0.046158) | 0.023111 (0.020629, 0.025710) | 0.053799 (0.048969, 0.058963) | 0.034226 (0.030026, 0.038715) | 6.87E-11 | 1.26E-07 | 5.10E-04 | 1.25E-04 |
| **Present Focus** | 0.127119 (0.120388, 0.132921) | 0.169680 (0.164156, 0.175435) | 0.142528 (0.135377, 0.148580) | 0.165319 (0.157966, 0.171782) | 1.30E-15 | 2.30E-05 | 3.30E-03 | 2.12E-01 |
| **Future Focus** | 0.013136 (0.011337, 0.014753) | 0.020847 (0.018956, 0.022743) | 0.016827 (0.015133, 0.018558) | 0.019308 (0.016615, 0.021985) | 2.46E-07 | 4.69E-01 | 3.32E-03 | 1.09E-01 |
| **Negative Emotionality** | 0.022703 (0.019987, 0.025372) | 0.013624 (0.011951, 0.015131) | 0.016879 (0.015043, 0.018677) | 0.014236 (0.012142, 0.016456) | 3.97E-07 | 1.53E-02 | 8.91E-04 | 8.32E-01 |
| **Positive Emotionality** | 0.036382 (0.033354, 0.039576) | 0.038636 (0.035793, 0.041496) | 0.028559 (0.026553, 0.030906) | 0.029216 (0.026495, 0.031958) | 3.48E-01 | 7.16E-01 | 3.40E-06 | 8.24E-06 |
| **Checking for Understanding** | 0.005176 (0.003737, 0.006810) | 0.003468 (0.002374, 0.004652) | 0.000206 (0.000070, 0.000397) | 0.000753 (0.000383, 0.001212) | 3.25E-01 | 3.72E-02 | 4.79E-12 | 3.83E-07 |
| **Demonstrating understanding phrases** | 0.000417 (0.000179, 0.000776) | 0.000602 (0.000358, 0.000882) | 0.000342 (0.000201, 0.000493) | 0.000128 (0.000045, 0.000213) | 9.78E-02 | 2.03E-02 | 2.88E-01 | 4.56E-03 |
| **Hedging** | 0.020788 (0.018675, 0.023064) | 0.022689 (0.020944, 0.024366) | 0.023633 (0.021048, 0.026423) | 0.028504 (0.025967, 0.031180) | 1.10E-01 | 9.04E-03 | 1.95E-01 | 3.89E-03 |
| **Absolutist** | 0.007045 (0.005710, 0.008628) | 0.007681 (0.006567, 0.008824) | 0.013815 (0.011383, 0.016654) | 0.009116 (0.007968, 0.010405) | 2.19E-01 | 7.48E-03 | 2.68E-07 | 4.44E-02 |
| **Seconds per talk turn** | 4.895247 (3.959944, 5.938566) | 7.161465 (5.676544, 9.161874) | 8.116844 (6.393563, 10.026997) | 4.874929 (4.210783, 5.614105) | 7.35E-04 | 5.60E-04 | 5.66E-05 | 9.42E-03 |
| **Seconds per talk turn ratio** | 0.938381 (0.766941, 1.133695) | 1.879465 (1.490541, 2.334864) | 0.938381 (0.766941, 1.133695) | 1.879465 (1.490541, 2.334864) | 4.95E-06 | 4.95E-06 | N/A | N/A |
| **Words per second** | 2.364974 (2.229546, 2.505802) | 2.585216 (2.448995, 2.726731) | 2.412626 (2.258546, 2.568551) | 2.337722 (2.195653, 2.483889) | 5.63E-02 | 1.99E-01 | 3.00E-01 | 2.19E-02 |
| **Words per second ratio** | 1.040044 (0.963403, 1.121408) | 1.171553 (1.096911, 1.253817) | 1.040044 (0.963403, 1.121408) | 1.171553 (1.096911, 1.253817) | 9.62E-03 | 9.62E-03 | N/A | N/A |

**Supplementary Table 1:** Comparison of language features between quintiles for therapist and patient. Values on the 4 leftmost columns represent average (95% CI) feature values, with confidence intervals generated via a percentile bootstrap (1000 bootstrap samples for each feature). Values in the four rightmost columns represent *p*-values resulting from running the Mann-Whitney U test, testing against the null hypothesis that language feature distributions from one partner/quantile are equal. More specifically, for two distributions *X* and *Y*, the Mann-Whitney U test tests against the null hypothesis that, taking a random sample from *X* and a random sample from *Y*, the sample from *X* is just as likely to be larger than *Y* as it is to be smaller than *Y*. Values highlighted in green are significant after controlling the False Discovery Rate at level α = 0.05 using the Benjamini-Hochberg procedure.

## Supplementary Table 2. Features considered but not selected for study

| **Construct** |
| --- |
| Therapist language that conveys hope |
| Therapist language that conveys warmth |
| Therapist asking for feedback—e.g., reactions to previous session; reactions during a session |
| Therapist facilitates a focus—a problem that both therapist and patient consider important |
| Therapist use of guided discovery (e.g., examining evidence) versus persuasion, debate |
| Therapist tracks and remarks on alliance rupture |
| Therapist addresses therapeutic rupture successfully |
| Helping achieve a change in appraisal e.g., reframing, “is there an alternative way of seeing this” (see Althoff et al. 2016: facilitating perspective change) |
| Therapist language that conveys action |
| LIWC categories for health/cognitive/relationships  (e.g., insight) |
| The Multitheoretical List of Therapeutic Interventions - 30 items (MULTI-30) -> n-grams from this scale |

**Supplementary Table 2:** Clinicians, using an iterative Delphi approach, generated a list of clinical features of interest that might be expressed and measured in language. Table 2 reports features considered, but not selected. Delphi phase of the study ended 4.26.2018.
