# Supplementary material for: A computational approach to measure the linguistic characteristics of psychotherapy timing, responsiveness, and consistency
Source: Npj Ment Health Res. 2022 Dec 2;1:19. doi: 10.1038/s44184-022-00020-9 (PMC10956022; doi:10.1038/s44184-022-00020-9)
Supplement: Supplementary file 3 — Reporting Summary [file 44184_2022_20_MOESM3_ESM.pdf]

nature portfolio

Corresponding author(s): Adam Miner

Last updated by author(s): Sep 5, 2022

Reporting Summary

Nature Portfolio wishes to improve the reproducibility of the work that we publish. This form provides structure for consistency and transparency in reporting. For further information on Nature Portfolio policies, see our Editorial Policies and the Editorial Policy Checklist.

Statistics

For all statistical analyses, confirm that the following items are present in the figure legend, table legend, main text, or Methods section.

n/a	Confirmed	
☐	☒	The exact sample size (n) for each experimental group/condition, given as a discrete number and unit of measurement
☐	☒	A statement on whether measurements were taken from distinct samples or whether the same sample was measured repeatedly
☐	☒	The statistical test(s) used AND whether they are one- or two-sided<br>*Only common tests should be described solely by name; describe more complex techniques in the Methods section.*
☐	☒	A description of all covariates tested
☐	☒	A description of any assumptions or corrections, such as tests of normality and adjustment for multiple comparisons
☐	☒	A full description of the statistical parameters including central tendency (e.g. means) or other basic estimates (e.g. regression coefficient) AND variation (e.g. standard deviation) or associated estimates of uncertainty (e.g. confidence intervals)
☐	☒	For null hypothesis testing, the test statistic (e.g. F, t, r) with confidence intervals, effect sizes, degrees of freedom and P value noted<br>*Give P values as exact values whenever suitable.*
☒	☐	For Bayesian analysis, information on the choice of priors and Markov chain Monte Carlo settings
☐	☒	For hierarchical and complex designs, identification of the appropriate level for tests and full reporting of outcomes
☐	☒	Estimates of effect sizes (e.g. Cohen's d, Pearson's r), indicating how they were calculated

Our web collection on statistics for biologists contains articles on many of the points above.

Software and code

Policy information about availability of computer code

Data collection	Psychotherapy transcripts were created from audio recordings of therapy sessions gathered per protocol from a randomized clinical trial. For details see: Miner, A.S., Haque, A., Fries, J.A. et al. Assessing the accuracy of automatic speech recognition for psychotherapy. npj Digit. Med. 3, 82 (2020). https://doi.org/10.1038/s41746-020-0285-8
Data analysis	All code was written in Python 3.8.5. For data preprocessing and statistical analysis, Pandas 1.2.0, NumPy 1.19.2, SciPy 1.5.2, SciKit-Learn 0.23.2, tigramite 4.2.1, networkx 2.5, statsmodels 0.12.1, and nltk 3.5 were used.

For manuscripts utilizing custom algorithms or software that are central to the research but not yet described in published literature, software must be made available to editors and reviewers. We strongly encourage code deposition in a community repository (e.g. GitHub). See the Nature Portfolio guidelines for submitting code & software for further information.

Data

Policy information about availability of data

All manuscripts must include a data availability statement. This statement should provide the following information, where applicable:

- Accession codes, unique identifiers, or web links for publicly available datasets
- A description of any restrictions on data availability
- For clinical datasets or third party data, please ensure that the statement adheres to our policy

Code for this study will be publicly available concurrent to publication. The datasets (i.e., psychotherapy transcripts) analyzed during the current study are not

publicly available due to the nature of personal disclosures in psychotherapy with the potential to compromise research participants' privacy, but datasets are available from the corresponding author on reasonable request and appropriate ethical oversight.

Human research participants

Policy information about studies involving human research participants and Sex and Gender in Research.

Reporting on sex and gender	Sex of participants was self-reported during the original clinical trial (for study details see: Wilfley DE, Agras WS, Fitzsimmons-Craft EE, et al. Training models for implementing evidence-based psychological treatment: A cluster-randomized trial in college counseling centers. JAMA Psychiatry. 2020;77(2):139-147.) The current study assessed differences based on sex based on prior findings in psychotherapy process research of sex-based effects.
Population characteristics	See below.
Recruitment	No recruitment happened for this study, as it was a secondary analysis of existing clinical trial data. For recruitment information of the original trial, see: Wilfley DE, Agras WS, Fitzsimmons-Craft EE, et al. Training models for implementing evidence-based psychological treatment: A cluster-randomized trial in college counseling centers. JAMA Psychiatry. 2020;77(2):139-147.
Ethics oversight	Study protocol was approved by Stanford University IRB.

Note that full information on the approval of the study protocol must also be provided in the manuscript.

Field-specific reporting

Please select the one below that is the best fit for your research. If you are not sure, read the appropriate sections before making your selection.

☐ Life sciences ☒ Behavioural & social sciences ☐ Ecological, evolutionary & environmental sciences

For a reference copy of the document with all sections, see nature.com/documents/nr-reporting-summary-flat.pdf

Behavioural & social sciences study design

All studies must disclose on these points even when the disclosure is negative.

Study description	This study is a secondary analysis of psychotherapy transcripts gathered per protocol during a completed multi-site randomized trial. The original study aim was to assess two distinct clinician training strategies. The current study, presented here, is a quantitative analysis of therapist and patient language patterns using natural language processing.
Research sample	Audio recordings of college counseling psychotherapy were gathered per protocol during the original clinical trial across 24 college counseling clinics in the United States. This research was blinded to site location and participant identities. It is unknown how well this sample generalizes to other counseling settings. The transcripts were generated from a HIPAA-compliant medical transcription company. In our sample, 87% of patients were female. Other demographic characteristics were blinded.
Sampling strategy	From the full sample of audio recordings, 100 were selected at random , with a sampling strategy that maximized the number of unique patients. Thus, there were 100 unique patient sessions sampled in this study. Some therapists saw more than one patient in the original study, and thus may be represented more than once in this sample. No therapist had more than three patients in this study sample.
Data collection	Audio was collected using handheld electronic audio recorders (a single recorder provided to each therapist per protocol) and saved as WAV or MP3 files. The open-source FFmpeg program was used to convert all audio files into a standard FLAC audio format. A third-party HIPAA-compliant transcription company completed human transcription of all audio files in to TXT file formats.
Timing	All psychotherapy sessions took place between April 2013 and December 2016.
Data exclusions	Two audio recordings were excluded because the patient-therapist dyad represented in the audio recording was already represented by a third audio recording included in the sample. Five samples were excluded from the analysis of therapist responsiveness. Three were excluded because one or more of the patient’s language features exhibited zero variance and thus were not amenable to the statistical procedures proposed. Two were excluded because their language patterns were nonstationary, a requirement for valid inference using our chosen statistical methodology.
Non-participation	This is a secondary analysis of psychotherapy transcripts; thus, there was no opportunity for non-participation. Study protocol was approved by Stanford University IRB.
Randomization	Randomization was not needed or appropriate for secondary analysis of psychotherapy transcripts.

Reporting for specific materials, systems and methods

We require information from authors about some types of materials, experimental systems and methods used in many studies. Here, indicate whether each material, system or method listed is relevant to your study. If you are not sure if a list item applies to your research, read the appropriate section before selecting a response.

Materials & experimental systems

n/a	Involved in the study
☒	☐ Antibodies
☒	☐ Eukaryotic cell lines
☒	☐ Palaeontology and archaeology
☒	☐ Animals and other organisms
☐	☒ Clinical data
☒	☐ Dual use research of concern

Methods

n/a	Involved in the study
☒	☐ ChIP-seq
☒	☐ Flow cytometry
☒	☐ MRI-based neuroimaging

Clinical data

Policy information about clinical studies

All manuscripts should comply with the ICMJE guidelines for publication of clinical research and a completed CONSORT checklist must be included with all submissions.

Clinical trial registration	n/a
Study protocol	n/a
Data collection	No data was collected for this study, as this was a secondary analysis of historical clinical data.
Outcomes	There were no clinical outcomes assessed in this study.
